# Editorial to “Impact of frailty in patients with non‐valvular atrial fibrillation undergoing catheter ablation”

**DOI:** 10.1002/joa3.13074

**Published:** 2024-05-30

**Authors:** Sayaka Kurokawa, Yasuo Okumura

**Affiliations:** ^1^ Division of Cardiology, Department of Medicine Nihon University School of Medicine Tokyo Japan

**Keywords:** catheter ablation, frailty, non‐valvular atrial fibrillation

Frail patients with non‐valvular atrial fibrillation (NVAF) are a growing population in super‐aged societies such as Japan, posing treatment and management challenges. Catheter ablation (CA) for atrial fibrillation (AF) is a widely accepted therapy that has recently been shown to improve symptoms, quality of life, and clinical outcomes compared to medical treatment. However, there is a need for discussion regarding whether CA is a treatment option that can be actively proposed for elderly people, especially frail elderly patients with AF. In this issue of the journal, Soejima et al.^1^ presented new insights into the improvement of frailty after CA in patients with NVAF.

There are skeptical views on the active indication of CA for AF in the elderly owing to the increased complications,^2^ and there have been concerns particularly negative opinions have been shown in the past for frail patients. In the United States, a retrospective study using Medicare fee‐for‐service billing codes has been conducted on the prognosis of frail patients who received CA for AF.^3^ Among the 5070 patients who received CA, 1955 were judged to be frail based on the Hospital Frailty Risk Score. The long‐term mortality rates (up to 630 days) in a group of patients with a mean age of 74.9 years increased as the frailty risk score increased. Restricted cubic spline regression analysis revealed an adjusted hazard ratio for long‐term mortality of 1.065 (95% CI: 1.054–1.077). Another retrospective study using the National Health Insurance Service claims database in Korea examined the therapeutic effects of CA and medication treatment in frail and non‐frail elderly patients with AF.^4^ Over a median follow‐up of 28 months, the risk of all‐cause death and composite outcomes including heart failure admission, stroke/systemic embolism, and sudden cardiac arrest were evaluated. While CA reduced the risk of these outcomes in non‐frail patients, it did not show a beneficial effect in frail patients. These findings suggest that clinicians should avoid invasive treatments such as CA when managing frail patients.

Soejima et al.^1^ conducted sub‐analysis of the RYOUMA registry,^5^ a multi‐center prospective observational study on perioperative and long‐term anticoagulation therapy management of CA in patients with AF in Japan, and yielded different results. They evaluated frailty in elderly patients who received CA for NVAF with a simple 5‐item frailty index and analyzed the outcomes of CA for each degree of frailty. Of 3027 patients in the RYOUMA registry,^5^ 203 who completed the 5‐item frailty index were analyzed. Among them, 26 patients (12.8%) were frail, 109 patients (53.7%) were pre‐frail, and 68 patients (33.5%) were robust. In all groups, the rate of freedom from AF recurrence up to 6 months was relatively good, with 88.5%, 91.7%, and 86.8% in the frailty, pre‐frailty, and robust groups, respectively. The strength of the study lay in its demonstration of improved frailty among patients with frailty or pre‐frailty after CA. In the frailty group, 2 (9.5%), 10 (47.6%), and 9 (42.9%) out of 21 patients were robust, pre‐frail, and frail at 6 months post‐CA, respectively. In the pre‐frailty group, 26 (30.2%), 52 (60.5%), and 8 (9.3%) out of 86 patients were robust, pre‐frail, and frail, respectively, at the same follow‐up point. The main factors considered for improving frailty after CA were improvements in weight loss, walking speed, and fatigue in the 5‐item frailty index. This result suggests that CA may have a beneficial therapeutic impact even in frail elderly patients with NVAF, which has been previously debunked.^3^, ^4^ In addition, the incidence of major bleeding, cardiovascular events, and cardiac events in the frail group was 0.3%/person‐year, 0.5%/person‐year, and 0.5%/person‐year, respectively, which were higher than in the other two groups, no cases of cardiovascular disease deaths, strokes/systemic thromboembolic events, or intracerebral hemorrhages were observed in any of the three groups.

However, it should be noted that this study was an analysis of a limited number of patients (*n* = 203) who answered the 5‐item frailty index among the patients registered in the RYOUMA registry.^5^ Furthermore, the number of patients who were judged to be frail before CA was very small (26 patients), and the number of cases was limited. Additionally, frailty comprises various elements. There may be variations in the group of patients with frailty extracted depending on the indicator used, and caution is required when interpreting the results. The average age and weight of patients in the frailty group in this study were 75 years, and the average weight is 56 kg suggests that the frailty judged by the 5‐item frailty index may have extracted a group with a relatively better overall condition than the super‐aged and low‐weight group that is a problem in actual clinical practice. Another aspect to consider is that the five items used for the diagnosis of frailty, walking speed, and fatigue may not necessarily indicate an improvement in frailty itself but rather an improvement in AF and the resulting heart failure owing to CA. Finally, enrollment criteria and frailty assessment in the registry may introduce bias from both physician and patient reporting. The potential influence of these biases on the study results should be addressed.

Despite the limitations, the study by Soejima et al.^1^ showed the importance of careful examination of the condition by clinicians to determine whether there are no improvable elements by intervention such as CA even in frail AF patients who had almost uniformly given up active treatment. In actual clinical practice, there are many cases where the management of AF becomes difficult even if only medication treatment is the selected treatment strategy in elderly frail patients owing to the deterioration of the overall condition due to the decrease in metabolic organ function, low body weight, and comorbidities. In addition, even in patients who seem to be frail at first glance, there may be cases where the fundamental cause of the symptoms judged to be frail is AF. This study is significant as it shows the possibility of expanding treatment options for frail elderly patients with NVAF, who were previously considered difficult to treat.

## FUNDING INFORMATION

None.

## CONFLICT OF INTEREST STATEMENT

Authors declare no conflict of interests for this article.
